# Erosive processes after tectonic uplift stimulate vicariant and adaptive speciation: evolution in an Afrotemperate-endemic paper daisy genus

**DOI:** 10.1186/1471-2148-14-27

**Published:** 2014-02-13

**Authors:** Joanne Bentley, G Anthony Verboom, Nicola G Bergh

**Affiliations:** 1 University of Cape Town, Cape Town, South Africa; 2 The Compton Herbarium, Kirstenbosch Research Centre, South African National Biodiversity Institute, Private Bag X7, Newlands, Cape Town, South Africa

**Keywords:** Afrotemperate, Drakensberg, Uplift, Adaptive speciation, Vicariance, Gnaphalieae

## Abstract

**Background:**

The role of tectonic uplift in stimulating speciation in South Africa’s only alpine zone, the Drakensberg, has not been explicitly examined. Tectonic processes may influence speciation both through the creation of novel habitats and by physically isolating plant populations. We use the Afrotemperate endemic daisy genus *Macowania* to explore the timing and mode (geographic versus adaptive) of speciation in this region. Between sister species pairs we expect high morphological divergence where speciation has happened in sympatry (adaptive) while with geographic (vicariant) speciation we may expect to find less morphological divergence and a greater degree of allopatry. A dated molecular phylogenetic hypothesis for *Macowania* elucidates species’ relationships and is used to address the potential impact of uplift on diversification. Morphological divergence of a small sample of reproductive and vegetative characters, used as a proxy for adaptive divergence, is measured against species’ range distributions to estimate mode of speciation across two subclades in the genus.

**Results:**

The *Macowania* crown age is consistent with the hypothesis of post-uplift diversification, and we find evidence for both vicariant and adaptive speciation between the two subclades within *Macowania*. Both subclades exhibit strong signals of range allopatry, suggesting that geographic isolation was important in speciation. One subclade, associated with dry, rocky environments at high altitudes, shows very little morphological and ecological differentiation but high range allopatry. The other subclade occupies a greater variety of habitats and exhibits far greater morphological differentiation, but contains species with overlapping distribution ranges.

**Conclusions:**

Species in *Macowania* are likely to have diversified in response to tectonic uplift, and we invoke uplift and uplift-mediated erosion as the main drivers of speciation. The greater relative morphological divergence in sympatric species of *Macowania* indicates that speciation in the non-sympatric taxa may not have required obvious adaptive differences, implying that simple geographic isolation was the driving force for speciation (‘neutral speciation’).

## Background

The formation of major mountain chains by tectonic uplift has stimulated plant diversification in many parts of the world, and the resulting diversity may be spectacular. Documented examples include the Andes [1-3], the Mexican Sierra Madre [4-6] and the Himalayas [7,8]. For example the northern Andes harbours some 45,000 plant species, 44% of which are endemic [9], with the northern Andean páramos topping this with 60% endemism [10]. However, the specific mechanisms by which uplift may influence species divergence have seldom been explicitly explored. In this study, we examine the mechanisms underlying speciation following uplift of a diverse South African mountain system.

**Figure 1:**
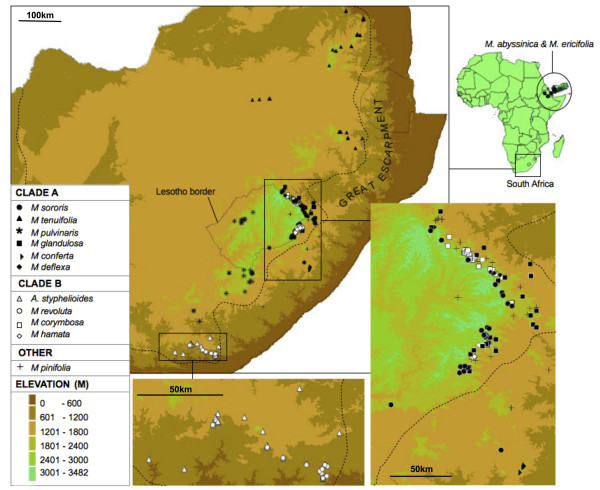
**Distribution map of *Macowania *and *Arrowsmithia*.** Clade A species are indicated by black symbols, clade B species by white symbols. The dotted line represents the Escarpment edge. The disjunct distributions of *M. abyssinica* and *M. ericifolia* in the East African highlands and Yemen are indicated at the top right of the figure. Insets of selected parts of the distribution in South Africa are provided for small areas that house multiple species.

In southern Africa, Pliocene tectonic uplift played a major role in creating the geomorphically diverse Drakensberg (‘Dragon’s Mountain’) range [11-14]. The Drakensberg constitutes the higher, eastern façade of the central plateau (‘the Great Escarpment’) of South Africa (Figure 1). In terms of both height and endemic plant diversity, the Drakensberg is more modest than the Andes, with a maximum altitude of 3,482 m and the core area hosting *ca* 2,520 species or subspecies of flowering plants, of which 16% are endemic [15]. Nevertheless, the Drakensberg region is one of three centres of Afrotemperate endemism, and appears to have been an important link between the centre in the Ethiopian highlands and that in the highly diverse Cape region with dispersal in both directions resulting from, and possibly facilitating, floristic radiations (e.g. northwards after radiation: *Disa*, Irideae, *Pentachistis*, Restionaceae [16]; southwards: *Scabiosa*[17]; *Erica*[18]). Within the mega-diverse southern African subregion, the Drakensberg constitutes the highest-lying land and the only true alpine habitat [19,20]. The endemic Drakensberg flora is thus directly or indirectly a product of the tectonic processes that created these high-altitude habitats.

Tectonic uplift might stimulate speciation in two principal ways. First, pronounced uplift will produce a novel, high-altitude adaptive zone which may serve as an arena for adaptive radiation (*sensu* Simpson [1]). The scale of radiation that occurs within such an adaptive zone will ultimately depend on the heterogeneity of habitats on offer, but this is likely to be high owing to the effectiveness of tectonism and subsequent erosion in generating situations that vary in terms of altitude, aspect, slope, geology, soil type, microclimate and moisture regime (e.g. stream beds versus rocky ridges). Underlying geological heterogeneity will increase the overall habitat diversity by allowing for specialisation to different soil types as they are exposed or created by erosion; it will also result in a more complex landscape due to differences in erodibility. Tectonism may also promote species radiation indirectly, by the stimulation of large-scale erosion resulting from increased river gradients. Removal of increasing portions of the landscape will disrupt the original land surface, potentially fragmenting species ranges. Given time, populations separated by erosion gaps will diverge as a consequence of both adaptive divergence and neutral processes (drift), the latter being more important where the selective differentials between populations are low and/or population size is small with limited gene flow [21]. An example of neutral divergence following erosion-mediated habitat fragmentation is the divergence of a montane lizard genus (*Phrynosoma*) in the Sierra Madre of Mexico, where canyon formation followed tectonic uplift [4-6]. Although the potential importance of non-adaptive divergence as a driver of speciation (non-ecological speciation) is gaining recognition [22-24], the long-standing emphasis on adaptive divergence as the sole agent remains pervasive [25-28], and it is difficult to prove the absence of any form of adaptation.

In principle, post-uplift species accumulation is likely to occur as a consequence of both adaptive and non-adaptive processes. If this is true, tectonic processes and subsequent erosion have likely been important as stimuli for both adaptive and non-adaptive diversification in the Drakensberg. One of the predictions of this hypothesis is that the bulk of diversification in endemic lineages should be associated with (or occur soon after) episodes of major tectonic activity. Also, where non-adaptive processes have been important, we expect to find strong signatures of allopatric speciation, paired with limited functional divergence. A brief overview of the geological history of the region reveals high probability of erosion-mediated range fragmentation. Composed of large blocks of sedimentary and volcanic rocks (primarily the soft ‘cave’ sandstones of the Clarens group topped by the younger and erosion-resistant basalts and dolerites of the Drakensberg group) which have been deposited over the past 200 Ma, the highly incised contemporary landscape of the Drakensberg is a product of erosion associated with cyclical uplift throughout the Cenozoic. Following the separation of Africa from the rest of Gondwanaland at about 184 Ma, the eastern half of South Africa experienced several cycles of uplift. The two most recent cycles, the first occurring in the early Miocene and the second in the early Pliocene (± 5 Ma), are thought to have raised the eastern margin of the Great Escarpment of southern Africa by between 150 – 300 m and 600 – 900 m respectively [12,14,29-31], each cycle stimulating renewed erosion [11-13]. Over time, the resulting escarpment edge, originally a uniform plateau extending to nearly the present-day coastline [11], is hypothesised by some authors [11-13] to have eroded back towards the interior of the country, simultaneously being incised by a series of deep drainage gulleys. The greater elevations of the Drakensberg range, relative to the rest of the Great Escarpment, has been attributed to pronounced upward flexing in response to to local intense erosion on one flank [32-34]. In the absence of historical volcanism, the contemporary deeply-dissected and geologically heterogeneous Drakensberg landscape is thus a product of landscape erosion.

The existence of the Drakensberg has undoubtedly been key to the creation of the ‘Afrotemperate track’ [20,35,36], a continuous zone of floristic affinity between the hyper-diverse Cape Floristic Region and the Afrotemperate regions of tropical Africa and the Mascarenes [37]. Dispersal both to and from the Cape via the Drakensberg may have been an important factor in the genesis of Afrotemperate plant diversity, including the floras of Madagascar and upland tropical Africa, as well as providing opportunities for European alpine lineages to disperse to and subsequently diversify in the Cape. Regional floras are assembled via both immigration and *in situ* diversification. Most Drakensberg-endemic lineages studied to date appear to be the result of repeated independent dispersal into the region, rather than *in situ* diversification [16]. Evidence for this lies in the low number of endemic species per genus. Of the 37 genera that contribute more than three endemic species to the Drakensberg Alpine Centre (DAC: the central, highest-lying part of the Drakensberg range [38,39]), only two have more than 12 endemics and the average is 6 endemic species [40]. Although the scale of *in situ* radiation varies among Drakensberg plant lineages [16] it appears for the most part to be modest, which may reflect both a youthful colonisation history and the small scale of the region. The daisy family Asteraceae has been the most successful angiosperm coloniser of the Drakensberg, with several genera contributing high numbers of endemic species to the DAC (*Helichrysum*: 29; *Senecio*: 22; *Euryops*: 7 [38]).

The paper daisy genus *Macowania* Oliver has five species strictly endemic to the DAC, but ten species endemic to the greater Drakensberg area (including the escarpment of the Eastern Cape, KwaZulu-Natal and Mpumalanga provinces). This makes it comparatively species-rich, and an excellent system for exploring the impact of landscape evolution on speciation in the greater Drakensberg area. In total, *Macowania* comprises 12 evergreen, woody sub-shrubs, the two non-Drakensberg species being native to the highlands of Ethiopia, Djibouti, Eritrea and Yemen (henceforth referred to as ‘East Africa’; Figure 1) and comprising the northern extent of the typical ‘Afrotemperate track’ [35]. The South African species of *Macowania* are essentially restricted to high-elevation habitats, the majority favouring rocky environments along or immediately below the Drakensberg scarp edge. Here they inhabit a diversity of substrata or geologies, and some degree of substrate-specificity is apparent. Three species are unusual in preferring riparian or frequently-moist habitats.

**Figure 2:**
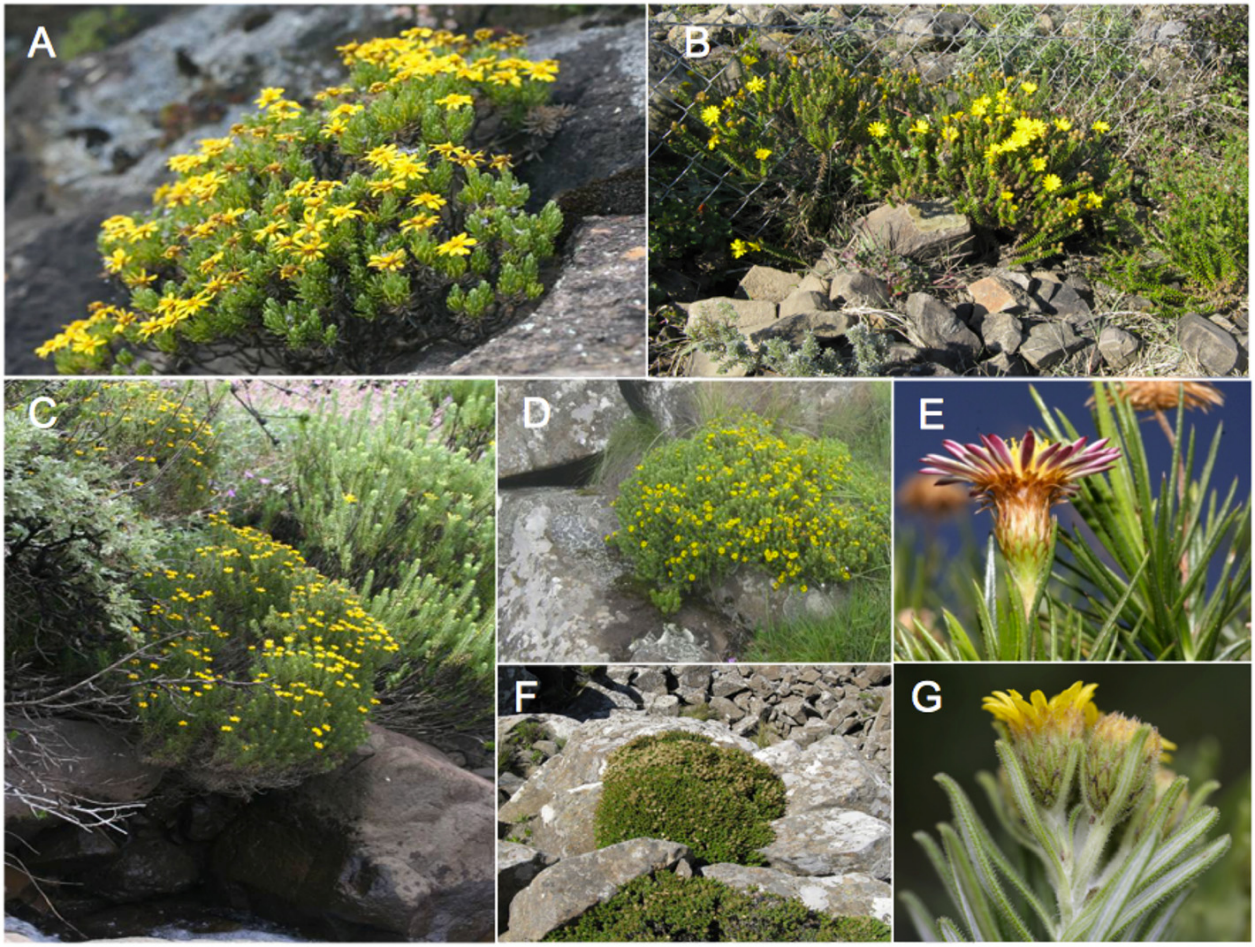
**Habitat, habit and morphology of selected study species. A**: *M. pulvinaris* (clade A)*. ***B**: *Arrowsmithia styphelioides* (clade B). **C**: *M. hamata*, overhanging a stream (clade B). **D**: *M. conferta* with cushioned growth form (clade A). **E**: *M. pinifolia*, unusual in *Macowania* due to its pink ray florets and non-revolute leaves. **F**: Habitat of *M. sororis* cushioned among dolerite rocks/boulders (clade A). **G**: Typical *Macowania i*nflorescence and leaves of *M. corymbosa* (clade A), also showing the dark-edged bracts shared by *R. revoluta* and *M. corymbosa.*

In this study, we present a dated molecular (nuclear and plastid DNA) phylogenetic hypothesis for *Macowania* and, in conjunction with distributional and morphological data, use this to explore speciation pattern and process. We include in our study the monotypic genus *Arrowsmithia* because, despite its contrasting vegetative morphology, past authors have suggested the possibility of a close relationship with *Macowania*[41,42]. In view of the overwhelming association of *Macowania* with high-elevation habitats, we hypothesise that the Drakensberg species of *Macowania* constitute a clade whose contemporary diversity is the product of a minor radiation associated with dramatic Pliocene uplift of the Drakensberg scarp. Our discovery within *Macowania* of two principal clades, one restricted to more-or-less uniform high-elevation rocky habitats (clade A) and the other occupying a broader array of ecological situations (clade B, occurring on rocky slopes, along streams and in seepages), indicates a potential role for both non-ecological and ecological speciation processes (Figures 1 and 2). We speculate that, whereas erosion-induced fragmentation of the scarp zone has resulted in a history of primarily vicariant speciation in clade A, speciation in clade B has been powered to a greater extent by adaptive divergence. To evaluate these ideas, we test the predictions that, consistent with a vicariant speciation model, (i) the signatures of allopatric speciation should be stronger in clade A than in clade B; (ii) morphological divergence, used as a proxy for functional diversification, should be less pronounced in clade A than in clade B; and (iii) where related species have highly overlapping distributions, especially in clade B, morphological divergence should be higher. We also use molecular dating to evaluate the hypothesis that radiation of these clades closely followed recent tectonic uplift at the start of the Pliocene.

## Methods

### Species collection and sampling

Between one and four accessions of all known species of *Macowania* and *Arrowsmithia* were sampled, resulting in a total of 34 ingroup individuals. We also included a single accession of each of the closely-related outgroup genera [43,44] (Table 1). More distant outgroups (*Galeomma* and *Ifloga*) represent early-diverging lineages of the ‘crown radiation’ of Gnaphalieae [45,46]. Leaf material was collected in the field and/or sampled from herbarium specimens from BOL, PRE, K, GRA or NU. While a large proportion of the available herbarium material yielded poor DNA, sequences from multiple accessions were nevertheless obtained for all species except *M. ericifolia* from East Africa (represented by a single accession).

**Table 1:** Sampling table of all species accessions used in this study

**Species name**	**Voucher details**	**GenBank accession number**				**Geographic origin**
	**Collector & herbarium**	**ITS**	**ETS**	**trnT-L**	**psbA-trnH**	
*Arrowsmithia styphelioides*	Bergh 2188 (NBG)	KF997177	KF997221	KF997136	KF997108	Mount Kempt
*A. styphelioides*	Bergh 2129 (NBG)	KF997178	KF997222	KF997135	–	Katberg Pass
*A. styphelioides*	O Hilliard & B Burtt 13266 (NU)	KF997179	KF997223	–	–	Katberg Pass
*Athrixia angustissima*	M Koekemoer 3550 (PRE)	KF997172	–	KF997131	–	Sehlabathebe National Park
*A. arachnoidea*	NG Bergh 2198 (NBG)	KF997173	–	KF997132	KF997105	Cathedral Peak
*A. elata*	NG Bergh 2203 (NBG)	KF997174	–	KF997133	KF997106	Pilgrims Rest
*A. fontana*	M Koekemoer 3554 (PRE)	KF997175	–	KF997134	–	Sehlabathebe National Park
*A. phylicoides*	NG Bergh 2180 (NBG)	KF997176	KF997220	–	KF997107	Cathedral Peak
*Comborhiza virgata*	NG Bergh 2174 (NBG)	KF997180	KF997224	KF997137	KF997109	Injusuthi
*Galeomma oculus-cati*	NG Bergh 1703a (NBG)	–	FR821616	FR821716	–	Western Cape
I*floga spicata*	J Lambinon 17590 (NBG)	–	FR821628	FR821728	–	Western Cape
Leysera leyseroides*	Lippert 22077 (PRE)	KF997181	–	KF997138	–	Morocco
Macowania abyssinica*	Friis et al. 12210 (K)	KF997182	–	KF997139	–	Ethiopia
M. abyssinica*	Polunin 11650 (K)	KF997183	–	–	–	Ethiopia
*M. conferta*	NG Bergh 2245 (NBG)	KF997184	KF997225	KF997140	KF997121	Mount Ngeli
*M. conferta*	NG Bergh 2246 (NBG)	KF997185	KF997226	KF997141	–	Mount Ngeli
*M. corymbosa*	J Bentley 002 (NBG)	KF997186	KF997227	KF997142	KF997115	Cathedral Peak
*M. corymbosa*	ARA Noel 1672 (GRA)	KF997188	–	–	–	Sinyazi
*M. corymbosa*	NG Bergh 2177 (NBG)	KF997187	KF997228	KF997143	KF997110	Injusuthi
*M. deflexa*	NG Bergh 2173 (NBG)	KF997189	KF997229	KF997144	KF997111	Injusuthi
*M. deflexa*	NG Bergh 2178 (NBG)	KF997190	KF997230	KF997145	–	Injusuthi
M. ericifolia*	Miller 3133 (K)	KF997191	–	KF997146	–	Yemen
*M. glandulosa*	NG Bergh 2181 (NBG)	KF997192	KF997231	KF997147	KF997112	Cathedral Peak
*M. glandulosa*	O Hilliard & B Burtt 17984 (NU)	KF997193	KF997232	–	KF997124	Sani pass
*M. hamata*	JP Roux 1826 (NBG)	KF997194	KF997233	KF997148	–	Sani Pass
*M. hamata*	NG Bergh 2166 (NBG)	KF997195	KF997234	KF997149	KF997113	Sani Pass
*M. hamata*	CJ Ward 10145 (PRE)	KF997196	KF997235	KF997150	KF997114	Sani Pass
*M. pinifolia*	J Bentley 003 (NBG)	KF997197	KF997236	KF997151	KF997116	Royal Natal Drakensberg
*M. pinifolia*	J Bentley 004 (NBG)	KF997198	KF997237	–	KF997117	Royal Natal Drakensberg
*M. pinifolia*	MP Robertson 74 (PRE)	KF997199	KF997238	KF997152	KF997118	Sani Pass
*M. pinifolia*	TD Abbot 7875 (PRE)	KF997200	KF997239	KF997153	KF997119	Garden castle forest reserve
*M. pulvinaris*	JE Victor 1569 (PRE)	KF997201	KF997240	KF997154	KF997123	Barkly East
*M. pulvinaris*	M Koekemoer 1581 (PRE)	KF997202	KF997241	KF997155	KF997122	Rhodes
*M. pulvinaris*	NG Bergh 2140 (NBG)	KF997203	KF997242	KF997156	–	Naudes Nek Pass
*M. revoluta*	J Bentley 001 (NBG)	KF997204	KF997243	–	–	Evelyn Valley Forestry Station
*M. revoluta*	J Bentley 005 (NBG)	KF997205	KF997244	KF997157	KF997120	Evelyn Valley Forestry Station
*M. sororis*	TR Green 1237 (NU)	KF997206	KF997245	KF997158	KF997126	Sani Pass
*M. sororis*	FK Hoener 1714 (NU)	KF997207	–	–	–	Sehlabathebe National Park
*M. sororis*	NG Bergh 2161 (NBG)	KF997208	KF997246	KF997159	KF997125	Mount Currie
*M. tenuifolia*	M Koekemoer 2079 (PRE)	KF997209	–	KF997160	KF997127	Mount Sheba
*M. tenuifolia*	M Koekemoer 2100 (PRE)	KF997210	KF997247	KF997161	KF997128	Mashishing
*M. tenuifolia*	NG Bergh 2211 (NBG)	KF997211	KF997248	KF997162	KF997129	Mount Sheba
*Oedera genistifolia*	NG Bergh 1572 (NBG)	KF997212	KF997249	KF997163	–	Grahamstown
*O. steyniae*	NG Bergh 1762 (NBG)	KF997213	KF997250	KF997164	–	Vermaaklikheid
*O. uniflora*	NG Bergh 1597 (NBG)	KF997214	KF997251	KF997166	–	Napier
Pentatrichia petrosa*	E Klaasen 2143 (WIND)	FR832509	FR823348	FR832580	–	Namibia
*Relhania acerosa*	NG Bergh 2137 (NBG)	KF997215	KF997252	KF997167	–	Naudes Nek Pass
*R. dieterlenii*	NG Bergh 2148 (NBG)	KF997216	KF997253	KF997168	KF997130	Rhodes
*R. rotundifolia*	T Oliver sn	KF997217	KF997254	KF997169	–	Riverlands Nature Reserve
*Rhynchopsidium sessiliflorum*	NG Bergh 2062 (NBG)	KF997218	KF997255	KF997170	–	Karoopoort
*Rosenia humilis*	M Koekemoer 2865 (PRE)	KF997219	–	KF997171	–	Victoria West

All samples were collected in South Africa, except those indicated by*.

### DNA extraction and sequencing

Total genomic DNA was isolated from silica-dried, field-sampled material using the CTAB extraction protocol of [47] modified according to [48], while the Qiagen DNeasy plant extraction-kit (Qiagen Sciences, Valencia, California, U.S.A.) was used for herbarium material. Two nuclear and two plastid regions with proven phylogenetic utility in Gnaphalieae were utilised [43,45,49]. The 3′ end of the external transcribed spacer (ETS) of nuclear ribosomal DNA was amplified using the primers 18S-ETS [50] and AST-1 [51] while the associated ITS1 and ITS2 introns and the intervening 5.8S ribosomal gene were amplified as a unit using the ITS4 and ITS5 primers of [52]. For the chloroplast genome, the *trnT-trnL* spacer was amplified using the primers ‘trna’ and ‘trnb’ of [53] and the *psbA-trnH* spacer was amplified with the trnH-R and psbA-F primers of [54].

PCR was performed in an Applied Biosystems 2720 thermal cycler (Applied Biosystems CA, USA) with the following thermal profile: initial denaturation of two minutes at 94°C; 35 cycles consisting of 94°C for 45 sec, 52°C for 45 sec (annealing) and 72°C for two min (extension); and a final extension step of 72°C for eight min. Reaction mixtures consisted of 12.8 μl nuclease-free H_2_O, 2.5 μl of 10x buffer (Kapa Biosystems Inc., MA, USA), 1.5 μl of 25 μM MgCl_2_, 1 μl dNTP mix at 0.2 μM each dNTP, 0.5 μl DMSO, 1.25 μl of each primer at 10 μM, 0.2 μl of Taq DNA polymerase (Kapa Biosystems Inc., MA, USA) and 4 μl of template DNA at various dilutions. Successfully amplified products were, for the most part, cleaned and sequenced by Macrogen (Macrogen Inc, Korea), who employed BigDye terminator cycling, using the amplification primers, with an ABI Automated Sequencer 3730XL being used to visualise the products (Life Technologies Corporation, Carlsbad, California, U.S.A.). Some products were, however, submitted to the Central Analytical Facility at Stellenbosch University, South Africa, where they were sequenced using a 3130XL Genetic Analyzer/3730 Genetic Analyzer. Chromatograms were assembled, examined and corrected where necessary using Geneious Pro v 5.4.4 (Biomatters Ltd., 2011) and manually aligned using BioEdit v 7.1.3.0 [55].

### Phylogenetic analysis

Indels were treated as missing data in all analyses. For the individual nuclear and plastid analyses the dataset was pruned to include only taxa that are represented by the relevant gene region. To check for topological incongruence, the four DNA regions were first analysed individually, and support for recovered clades evaluated using the parsimony bootstrap [56] in PAUP v 4.0 [57]. Although subject to dataset-specific biases and potentially problematic under certain conditions (e.g. long-branch attraction, lack of support on short internodes: [58,59]), the bootstrap is a relatively conservative measure of topological support [60]. One thousand bootstrap replicates were performed using only parsimony-informative sites, with tree bisection-reconnection (TBR) branch swapping on 100 random-addition trees with the multrees option not in effect and saving 100 trees per random-addition replicate. Trees were rooted on *G. oculus-cati* and *I. spicata.* Individual bootstrap consensus trees were examined for conflicting nodes supported by bootstrap percentages of 75% or higher. Since no such nodes were found, the datasets were concatenated, and a parsimony analysis of the combined data executed with the same settings.

The combined data (including those taxa represented by only nuclear or only plastid data) were also analysed using Bayesian phylogenetic inference, as implemented in MrBayes v 3.1.2 [61]). For this purpose, MrModeltest v 2.2 [62] was used to determine the optimal available models of DNA evolution under the AIC criterion [63]. This identified the GTR + G model as optimal for ITS, the GTR + I + G model for ETS and the GTR model for both plastid regions. A mixed model approach was employed in which substitution model parameters were estimated separately for each of three data partitions: (i) ETS, (ii) ITS and (iii) a combined plastid partition (all genes in the chloroplast genome are linked and should share the same phylogenetic history) using a Metropolis-coupled Markov Chain Monte-Carlo (MCMCMC) sampling procedure. Two concurrent analyses were run for 10^7^ generations each, starting with a different random tree and with parameters being sampled every 1,000 generations. The chain heating parameter was set at 0.3 and apart from the model settings, the default settings were retained. This analysis was repeated three times resulting in an overall total of six independent runs.

Convergence and stationarity were examined using the average standard deviation of split frequencies as output by MrBayes. The tree topologies from the six independent runs were also compared to check whether the runs were converging on the same topology. Convergence was further tested in Tracer v 1.3 [64] where the parameter estimates, ESS scores and likelihood traces were examined. Using the above checks, we discarded the first 10% of samples from each run as burn-in.

### Estimation of lineage divergence times

Divergence times were estimated using an uncorrelated relaxed lognormal Bayesian clock as implemented in BEAST v 1.6.2 [64], the input data being configured using BEAUTi v 1.6.1 [64] (BEAST .xml file available on request from the corresponding author). A paucity of fossil data renders molecular clock calibration difficult in Asteraceae, forcing us to employ a secondary calibration procedure. For this purpose, we made use of two nodes dated by Bergh and Linder [40] (Nodes B [Gnaphalieae crown age] and K [*Relhania* clade crown age]). To account for the compounding of error associated with secondary calibration (e.g. [65]), the priors on the two calibration nodes were specified in such a ways as to incorporate the uncertainty associated with Bergh and Linder’s [40] posterior age estimates. In each case, this was done by setting the mean and 95% CI of the normal prior to be equal to the mean and 95% HPD estimates reported by Bergh and Linder. The relaxed clock analysis employed a Yule tree prior and a mixed-model approach in which the sequence partitions and their associated models of nucleotide substitution were specified as for the MrBayes analysis. Two MCMC chains were run for 3 × 30^7^ generations each, with sampling every 1,000 generations. The results of these runs were tested for convergence as described above, and the runs were combined using LogCombiner v 1.6.2, again discarding the first ten percent of each sample as burn-in. The maximum clade credibility tree, with median node ages, was then extracted using TreeAnnotator v 1.6.2 [64].

### Range overlap

To measure pairwise range overlap, species’ ranges were estimated by plotting point locality data and calculating convex hull polygons. The localities of all relevant specimens at five South African herbaria (NBG, BOL, PRE, PRU, NU), as well as field observations by the authors were geo-referenced as precisely as possible using 1:50,000 topographic maps (Chief Directorate: Surveys & Mapping, Mowbray, Cape Town) in an ArcMap 10 environment (ArcGIS Desktop 10 Service Pack 2: CA: ESRI), as well as Google Earth and Google Maps. Where recorded, GPS co-ordinates provided a precise indication of locality. Convex hull polygons (i.e. the polygon that would be created by placing a tightly-stretched elastic band around all the point localities for a species) were produced using the ‘clusthr’ command in the ‘adehabit’ package [66] in R v 2.15.1 (R Development Core Team 2008). Convex hulls yield simplistic estimates of species’ ranges, ignoring range discontinuities and irregularities in range boundaries. Nonetheless, they are likely to closely approximate the ranges of species that have compact, continuous distributions, as is typical for *Macowania* and *Arrowsmithia.* In addition, the fact that this method over-estimates the extent of geographic ranges renders it a conservative measure of allopatry, making it robust for our purpose. Since convex hulls are sensitive to spatial errors [67], every effort was made to check and correct specimen identifications and locality information, and doubtful localities were excluded. Nevertheless, for several taxa very few precise localities were available and we had to use some observations that we deemed were accurate only to the nearest 5,001 – 10,000 m, the minimum number of locality points being six for the local endemic *M. deflexa*. Once the polygons were defined, the nested average of range overlaps between species was calculated as per Fitzpatrick and Turelli [68] using the BEAST tree topology, trimmed of multiple species accessions.

### Testing for adaptive differentiation

Adaptive divergence in montane settings could be driven by a range of selective forces, acting alone or in combination (for example, specialisation to particular climatic or edaphic niches or to different pollinators). As a proxy for adaptive differentiation, we looked for a suite of vegetative and reproductive characters that were relatively uniform within species of the core *Macowania* clade, but able to fairly reliably differentiate amongst species. Our rationale was that characters showing such patterns are likely to be under selection, or linked to other traits that are under selection. The characters selected were capitulum length and width (relating to reproduction, including floral display, seed size and number and seed protection) and leaf length and width (shown to be strongly correlated with plant habitats [69]). Precision callipers were used to measure 20 specimens per species, choosing representatives from across the geographic range of each. Owing to limited numbers of herbarium specimens, fewer measurements were taken for the localised endemics *M. deflexa* (two specimens), and *M. conferta* (six), as well as for *M. hamata* (14) and *M. revoluta* (18). For each character, the measurements from all specimens of a species were averaged and input into a pairwise multivariate discriminate functions analysis (DFA), as implemented in R v 2.15.1. Mahalanobis’ [70] distance, widely used in biological clustering, was then calculated between species. Mahananobis’ distance uses both the mean and variance of the predictor variables, as well as the covariance matrix of the variables, thus taking advantage of the covariance among variables. By transforming measurements into standardised uncorrelated data which is used to estimate Euclidean distances, scale differences are taken into account when estimating distances.

## Results

### Phylogenetic relationships in *Macowania*

**Figure 3:**
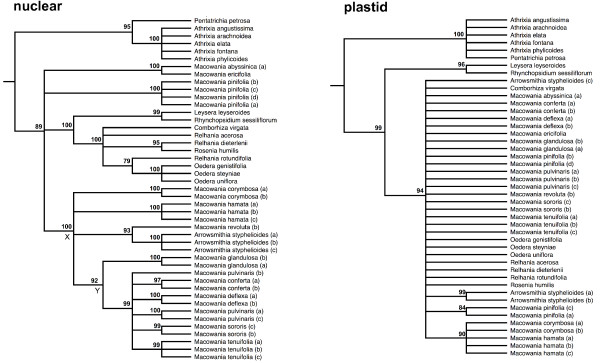
**Separate nuclear (combined ITS and ETS) and plastid (combined *trnT-trnL *and *psbA-trnH*) 75% majority-rule consensus parsimony topologies from PAUP.** Bootstrap values are indicated above the branches. Node ‘X’ and node ‘Y’ in the nuclear tree are referred to in the text. Multiple accessions of each species are distinguished by lower-case letters after the species name (see Table 1). Gnaphalieae outgroup specimens *Galeomma* and *Ifloga* have been trimmed from the tree.

The two nuclear regions (ETS & ITS) produced completely congruent trees, and so were combined to form a single nuclear matrix consisting of 47 accessions and 1,092 aligned nucleotides, of which 433 (40%) characters were parsimony-informative (Figure 3). Similarly, the two plastid regions (*trnT-L* and *psbA-F*) yielded poorly-resolved but congruent trees, and were combined to form a matrix consisting of 1,051 characters, of which 104 (10%) were parsimony-informative (Figure 3). Both the plastid and the nuclear gene trees independently recover *Macowania* as part of a clade containing the *Relhania* group of genera (represented here by *Relhania, Oedera* and *Comborhiza*). While the plastid topology neither rejects nor confirms the monophyly of *Macowania,* the nuclear gene tree resolves a clade comprising most species of *Macowania* (Node X; bootstrap percentage (BS) = 100) and including *Arrowsmithia.* Within this clade, a group of *Macowania* species form a strongly supported subclade (Node Y; BS = 99). Multiple accessions of species were always recovered as monophyletic in the nuclear ribosomal tree, while only those from *Arrowsmithia* and *M. pinifolia* grouped together at the 75% BS level in the plastid tree. Conflict between nuclear and plastid partitions is observed only with regard to the placement of the outgroup taxa *Leysera leyseroides* and *Rhynchopsidium sessiliflorum*. Since relationships amongst the ingroup taxa showed no conflict, all genetic partitions were concatenated into a single matrix and analysed in combination.

**Figure 4:**
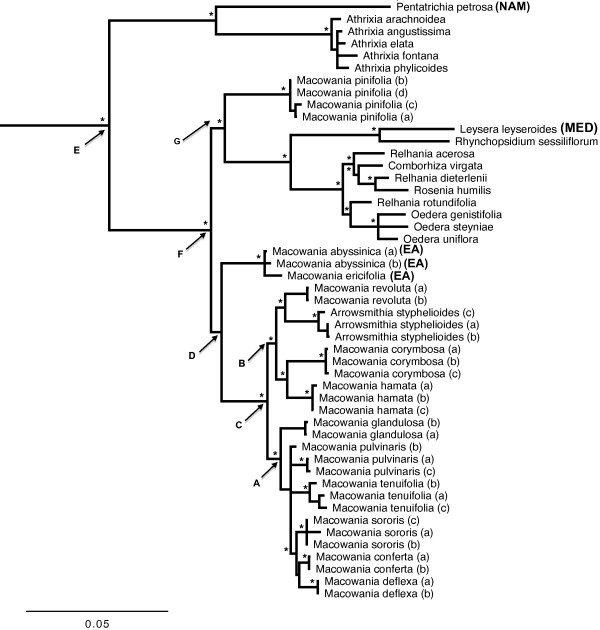
**Combined nuclear (ITS and ETS) and plastid (*trnT-trnL *and *psbA-trnH*) 50% majority-rule consensus Bayesian tree from the MrBayes analysis.** Nodes supported by either parsimony bootstrap (≥75%) *or* both Bayesian posterior probability (≥0.95) *and* BEAST posterior probability (≥0.95) are indicated with * above the relevant node. Note that node G is supported by Bayesian PP but not by BEAST PP). The support values for individual nodes A – G are reported in the text. Multiple accessions of each species are distinguished by lower-case letters after the species name (see Table 1). All species occur in South Africa or Lesotho (see Figure 1) except those with upper-case letters after the species: NAM (Namibia), NA (North Africa and/or Mediterranean and surrounds), EA (East Africa and Yemen). Gnaphalieae outgroup specimens *Galeomma* and *Ifloga* have been trimmed from the tree.

The combined plastid and nuclear tree is well-resolved with a topology that closely resembles the individual nuclear topology (Figure 4), with no observed decrease in support values upon the inclusion of taxa sampled for only nuclear or plastid data. While the monophyly of the *Relhania* clade *sensu* Bergh & Linder [43] is supported (Node E; MrBayes posterior probability (PP) = 1.0, BS = 100; hereafter referred to as the “*Relhania* clade *sensu lato*”), the strength of this result is compromised by the rather limited outgroup sampling. The monophyly of the *Relhania* clade *sensu lato* has, however, been verified in other studies with more extensive outgroup sampling [43,44]. There is also support for a clade consisting of *Macowania, Arrowsmithia, Relhania, Oedera, Leysera, Rhynchopsidium, Comborhiza* and *Rosenia* (henceforth named “*Relhania* clade *sensu stricto*”: Node F; PP = 1.0, BS = 100). Within the *Relhania* clade *sensu stricto, M. pinifolia* is placed as sister to a clade also comprising *Relhania, Oedera, Leysera* and relatives which is resolved as sister to the rest of *Macowania*. The placement of *Macowania pinifolia* in this position, however, lacks bootstrap support (Node G; PP = 0.97, BS < 75). Though the position of the East African species within *Macowania* is unsupported, these are nevertheless confirmed as most closely-related to *Macowania* than to any other genus (their inclusion in the genus has also been confirmed by bootstrap, Bayesian and BEAST PP support in a subsequent analysis where near-complete sampling with multiple species accessions of the *Relhania* clade *sensu lato* and additional outgroups has been carried out by Bentley et al. unpubl. data). The South African members of *Macowania* are monophyletic (Node C; PP = 1.0, BS = 100) subject to the inclusion of *A. styphelioides.* Within this ‘core’ *Macowania* clade, there is good support for two principal subclades, A and B. Clade B, which lacks support in the separate analyses, comprises *M. revoluta* (the type species)*, M. hamata, M. corymbosa* and *A. styphelioides* (PP = 1.0, BS = 97), while clade A (PP = 1.0, BS = 90), which was also recovered in the nuclear gene tree, comprises *M. tenuifolia, M. glandulosa, M. pulvinaris, M. deflexa, M. sororis* and *M. conferta*. The species relationships within clade A are largely unresolved, but there is good support (PP = 1.0, BS = 99) for a subclade containing *M. conferta, M. deflexa* and *M. sororis.* The monophyly of multiple accessions of each species in our tree is well-supported, with the exception of *M. pulvinaris* whose monophyly is not, however, contradicted.

### Divergence times and range overlap analysis

At least in terms of supported nodes, the relaxed clock analysis (Table 2; BEAST tree provided in Additional file 1) yielded the same topology as the MrBayes analysis, with strong support for most nodes. The *Relhania* clade *sensu lato* and *Relhania* clade *sensu stricto* (Nodes E and F: [43]) both have high support (PP = 1.0). The positions of *M. pinifolia* and the East African *Macowania* species (Node D), however, remain unresolved (PP < 0.95, BS < 75). The core *Macowania* clade is well-supported (PP = 1.0), as are clades A (PP = 1.0) and B (PP = 0.99). Within clade A, the BEAST topology differs slightly from that produced by MrBayes, specifically with regard to the placement of *M. tenuifolia*. This species is placed sister to the rest of the members of clade A in BEAST, whereas MrBayes favours *M. glandulosa* in this position, although with no support. Internal relationships do not affect subsequent analyses as these rely solely on species membership of clade A and B, not on their internal topologies.

**Table 2:** **BEAST median age estimates and 95% highest posterior density (HPD) in millions of years before the present for nodes A – F (see Figure **4**)**

**Node**	**Clade**	**Median**	**95% HPD**	**PP**
A	Clade A	3.6	1.20 – 7.00	1.00
B	Clade B	4.2	1.51 – 7.87	0.99
C	*Macowania* crown age	5.5	2.07 – 10.21	1.00
D	East African *Macowania* divergence	9.4	3.79 – 16.22	0.93
E	*Relhania* clade *sensu lato*	19.3	9.87 – 29.10	1.00
F	*Relhania* clade *sensu stricto*	8.5	3.70 – 14.49	1.00

PP = posterior probability of the node in the BEAST analysis.

Respectively, the median crown ages of the *Relhania* clade *sensu lato* (Node E) and *Relhania* clade *sensu stricto* (Node F) are estimated at 19.3 (95% HPD 9.9 – 29.1) Ma and 8.5 (3.7 – 14.5) Ma (Table 2). Although poorly supported in all analyses, the node indicating the divergence of the East African *Macowania* species from the core *Macowania* clade (Node D) is dated to 9.4 (3.8 – 16.2) Ma. The median crown age of the core *Macowania* clade (Node C) coincides with the Miocene/Pliocene boundary (5.5 Ma; 2.1 – 10.2 Ma), while those of clades A (Node A: 3.6 Ma, 1.2 – 7.0 Ma) and B (Node B: 4.2 Ma, 1.5 – 7.9 Ma) are both of Pliocene age, the error bars extending from the Late Miocene to Pleistocene. However, both dates have wide error margins which extend into the Miocene, indicating substantial uncertainty relating to their association with Pliocene uplift.

**Figure 5:**
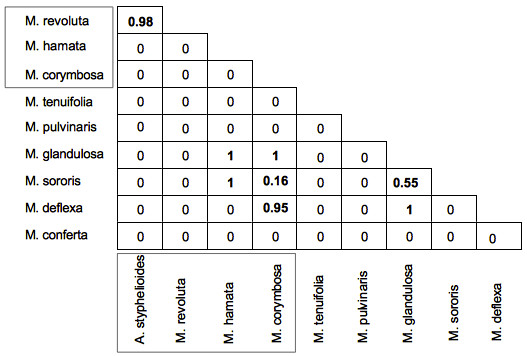
**Proportions of range overlap between species calculated using distributions estimated by convex-hull polygons.** Comparisons which involve overlapping ranges (i.e. all non-zero values) are indicated in bold; a value of 1 indicates comparisons in which the range of the more narrowly-distributed species is completely embedded within that of the species with the larger range. Species names surrounded by the boxes belong to clade B of the ‘core *Macowania* clade’.

Within *Macowania,* most species pairs exhibit zero range overlap (Figure 5). Of the eight comparisons which do show overlap, only three involve species from the same subclade (*M. revoluta* with *A. styphelioides* [clade B], and *M. glandulosa* with both *M. sororis* and *M. deflexa* [clade A]). The overlaps involving *M. glandulosa* may partly be a function of using convex hulls, since both species have crescent-shaped ranges (Figure 1). Moreover, where *M. glandulosa* generally favours sandstone substrates at lower altitudes, the latter two associate with the basaltic substrates of the high scarp (Figure 1), such that the true levels of sympatry between these species pairs may be negligible. The same is not true for *M. revoluta* and *A. styphelioides* which have been observed to co-occur at a number of localities (Figure 1), in wet and dry micro-habitats respectively. A comparison of the proportion of pairwise range overlaps between clades A and B revealed no significant differences between clades (t = -0.3885, df = 19, P > 0.05), signalling that range overlap levels are uniformly low in both clades.

### Potential adaptive differentiation

**Figure 6:**
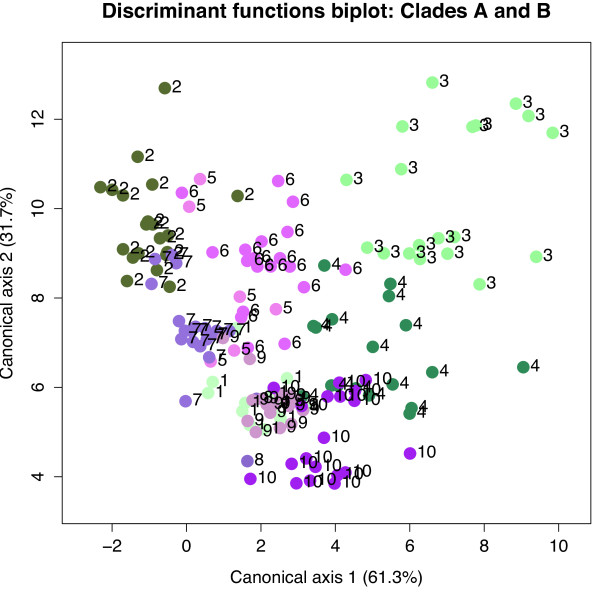
**Discriminant functions analysis biplot of Mahalanobis distances based on capitulum and leaf measurements.** Clade A species are indicated by symbols in shades of purple and pink while clade B species symbols are coloured shades of green. 1 = *M. hamata,* 2 = *A. styphelioides,* 3 = *M. corymbosa,* 4 = *M. revoluta,* 5 = *M. conferta,* 6 = *M. glandulosa,* 7 = *M. sororis,* 8 = *M. deflexa,* 9 = *M. pulvinaris* and 10 = *M. tenuifolia*.

Leaf and capitulum measurements provide a strong degree of morphological discrimination between species from clade B (symbols in shades of green), as indicated on the DFA biplot in Figure 6. Most members of clade B, however, show some degree of overlap with one or more species from clade A (symbols in shades of purple and pink), and all clade A species overlap with at least one other member of their clade. Based on the traits examined, species in clade B thus exhibit greater morphological divergence than those in clade A. Consequently, the Mahalanobis’ distances between species pairs within clade A are generally lower than those within clade B, and this difference is significant (t = 4.625, df = 14, P < 0.001) when compared against a randomly generated null. Of the 12 pairwise Mahalanobis’ distance comparisons within clade A, all have values of 100 or less, with only three (the comparisons of *M. glandulosa* with *M. deflexa*, *M. pulvinaris* and *M. sororis*) being greater than 30. In contrast, all but one comparison within clade B yield distances greater than 100, and the highest value is 900; clearly morphological divergence in the traits of interest is much higher within this clade.

## Discussion

To our knowledge, this study presents the first dated molecular phylogenetic study on a Drakensberg-near-endemic plant lineage as well as the first examination of geographic mode of speciation for the region. Phylogenetic data are fundamental to the study of lineage diversification, not only because they provide a (albeit tentatively) dated record of the successive speciation events underlying present-day species diversity, but also because they identify the bounds and membership of the lineage under study. Our data reveal *Macowania* to be non-monophyletic as currently circumscribed and, as such, inappropriate as a unit for evolutionary study. This is rectified, however, by the inclusion of the monotypic *Arrowsmithia* and the exclusion of *M. pinifolia*. Miocene-Pliocene uplift of the Drakensberg appears to have strongly influenced diversification within the *Macowania-Arrowsmithia* clade, because a strong signal of range allopatry points to geographical isolation as a key driver of speciation, and this diversification appears to have occurred post-uplift. We suggest that geographical isolation in *Macowania* is linked to Pliocene uplift and subsequent landscape erosion, these processes providing the necessary impetus for both non-ecological and ecological speciation.

### Taxonomic implications

The current circumscription of *Macowania* does not reflect evolutionary relationships because the genus is paraphyletic if *Arrowsmithia* is excluded, and *M. pinifolia* is not found to be a member of the genus. *Arrowsmithia* is strongly supported as a member of the ‘core’ *Macowania* clade which includes the type, *M. revoluta. Arrowsmithia* and *Macowania* differ in several morphological features, interpreted as key generic characters by previous taxonomists [41,42]. *Arrowsmithia* has shorter, broader leaves than core *Macowania* members, with only slightly revolute margins and without a thickened, raised abaxial midrib. In contrast, all core *Macowania* species have linear leaves with strongly revolute margins and a substantially raised and thickened abaxial midrib, giving the leaf a characteristic channelled appearance. At first glance, the capitula of *Arrowsmithia* are identical to those of *Macowania* (Figure 2), but Hilliard & Burtt [42] noted that the ray-floret achenes of *Arrowsmithia* have 20 ribs, compared with only 10 ribs in other members of *Macowania.* The only exception is *M. revoluta* which has 15 ribs [42] and was recovered as the sister to *Arrowsmithia* in our analysis.

A reconsideration of generic limits within the *Macowania* alliance is clearly required, and further questions relating to the monophyly of *Macowania* concern the status of the two East African species, *M. abyssinica* and *M. ericifolia*, as well as that of South African *M. pinifolia*. Although support for the relationship is poor, our analyses resolve *M. abyssinica* and *M. ericifolia* as sister to the ‘core’ *Macowania* clade (further substantiated by Bentley et al. unpubl. data), provisionally justifying their inclusion in the genus. In contrast to the situation for the East African species, the current data provide evidence against the continued inclusion of *M. pinifolia* in *Macowania* (supported by Bayesian PP, but not bootstrap or BEAST PP: Node G)*.* This is congruent with the assessment of Hilliard & Burtt [42], who suggest, mainly on the basis of leaf characters, that *M. pinifolia* is not closely related to the remainder of *Macowania.* Due to the limited *Relhania* clade outgroup sampling, a discussion of the relationships and non-monophyly of these lineages will be reserved for a future publication.

### Biogeographic history of *Macowania* in East Africa

A likelihood-based ancestral area biogeographic reconstruction based on a phylogenetic hypothesis produced using multiple species accessions and near-complete species sampling (Bentley et al. unpubl. data) indicates that the *Relhania* clade *sensu lato* originated in southern Africa. The timing of diversification in *Macowania*, and its exclusive occupation of Afrotemperate habitats, suggests that dispersal into East Africa was recent, probably following Miocene uplift [15]. The dates are similar to estimates for Drakensberg – East African migrations in *Disa*[16] but earlier than those estimated for *Euryops*[71]. One interpretation by our data is that the occurrence of *Macowania* at altitudes above 2,300 m in the greater northern Ethiopian highlands (Ethiopia, Eritrea and Yemen) might be the consequence of a northward dispersal around 10 Ma from a southern African centre of origin (though the error bars suggest substantial uncertainty in this estimate). Dispersal features in *Macowania* are weakly developed, consisting of small, light cypselas and a weak, feathery pappus with no specialised features to promote long-distance dispersal, and so species are likely to have low pappus-facilitated dispersibility. This, combined with the strict association of this genus with high altitudes and its apparent low dispersibility within South Africa, where most species are extremely localised, makes an expansive northward migration in *Macowania* an interesting prospect to unravel.

Uplifting of the East African Rift system started during the Eocene-Oligocene, attaining its greatest elevation during the Plio-Pleistocene interval [72-74]. One explanation for the East African disjunction in *Macowania* is that the topography of the high-altitude eastern leg of Africa was more uniform prior to this major uplift, facilitating northward dispersal. There is also a probable role for climate, with some authors [75-77] suggesting that wetter conditions prevailed along the eastern axis of Africa around 10 Ma, the onset of increased aridity and grassland expansion occurring later, at the Plio-Pleistocene boundary. If the ancestor of *Macowania* occupied multiple peaks along the eastern axis of Africa, then there would have been greater opportunity for the northeastward movement during wet periods, these peaks functioning as ‘stepping stones’ [16]. Subsequent aridification likely precipitated the extinction of the intervening populations, resulting in the isolation of the South African and East African populations, while Plio-Pleistocene changes in river systems [78] may also have played a role*.* Sustained isolation would have led to evolutionary divergence between the East African lineage and the ancestor of the ‘core *Macowania* clade’, the latter subsequently diversifying in the Drakensberg region.

### Pliocene uplift in the Drakensberg

Our data are consistent with the scenario of a Drakensberg radiation in *Macowania* in response to recent tectonic uplift. Firstly, there is strong support for the monophyly of a clade of Drakensberg-endemic taxa (the ‘core’ *Macowania* clade), indicating a single radiation here. Secondly, our BEAST analysis dates the radiation of this clade to shortly after the major Pliocene uplift of the eastern Escarpment [11-14], its crown node age being estimated at 5.5 Ma, though this estimate carries wide error margins (2.1 – 10.2 Ma). Our dates are, however, in line with earlier estimates of Drakensberg dispersals and radiations in *Pentaschistis, Disa* and *Morea*[16]. The bulk of the present-day species diversity is likely to have been generated more recently, with the crown nodes of the two principal subclades being dated to 3.6 Ma (clade A: 1.2 – 7.0 Ma) and 4.2 Ma (clade B: 1.5 – 7.9 Ma), consistent with a role for post-uplift scarp erosion.

Both subclades exhibit strong signals of range allopatry, suggesting that geographic isolation was important in speciation. Consistent with this idea, *Macowania* lacks specialised features which promote long-distance dispersal. Pronounced allopatry, in which distribution breaks coincide with erosion barriers, supports the idea that scarp erosion played a key role in the diversification of *Macowania.* This is best exemplified by the clade comprising *M. sororis*, *M. deflexa* and *M. conferta*. Species in this lineage consistently associate with high-altitude environments, all three being allopatric and for the most part very narrowly distributed. *Macowania conferta* is restricted to Ngeli Mountain, an isolated peak situated on the coastal plain about 85 km southeast of the main Drakensberg massif. Like Mount Currie, which supports the southeastern-most population of *M. sororis*, Ngeli is a relict fragment of a once-more extensive Drakensberg Escarpment, which has resisted the erosive forces that caused the Escarpment to retreat away from the present-day coastline. As such, the presence of *Macowania* on these peaks is likely also relictual, the disjunct nature of this distribution promoting vicariant divergence. Similarly, the deeply-incised Orange River canyon (Figure 1), the magnitude of its drainage accentuated by the increased westward tilt that Pliocene uplift conferred on the region [12], might explain the isolated and vicariant presence of *M. pulvinaris* on the opposing side of the river from other *Macowania* members, in western Lesotho and the northern reaches of the Eastern Cape Drakensberg. The presence of *M. tenuifolia* on isolated patches of Afrotemperate habitat in the Mpumalanga, Gauteng and Limpopo provinces might also be attributed to landscape erosion enabling divergence in allopatry. There are several floral elements linking this region with the KwaZulu-Natal Drakensberg (e.g. *Helichrysum subglomeratum, Selago procera*) as well as with the escarpment to the north in the Zimbabwean highlands (e.g. *H. swynnertonii, Aloe modesta;*[79]), perhaps suggesting the historical connection of this land.

### Speciation of *Macowania* in the Drakensberg

In the context of erosion-mediated vicariance, divergent selection may be critical in powering speciation (ecological speciation) or, somewhat more controversially [24,26], speciation may be powered by neutral processes alone (non-ecological speciation). Weak habitat and morphological differentiation between species within clade A suggest a primary role for isolation in species formation (non-ecological speciation). This is particularly evident for *M. sororis, M. deflexa, M. conferta* and *M. pulvinaris*, which exhibit no range overlap and very little morphological divergence, at least in terms of the traits sampled in this study. Taxonomically, these species are distinguished only by subtle characters of the peduncles and leaf-glands [80].

There is, however, some evidence for ecological divergence within clade A. Although *M. glandulosa* is broadly sympatric with *M. sororis* and *M. deflexa* (the only instances of sympatry in this clade), it shows some evidence of fine-scale ecological differentiation. Where the latter species inhabit scarp edge basalt substrates, *M. glandulosa* favours lower elevations, growing on the sandstone platforms that underlie the basalts [80]. Associated with this habitat shift is a morphological transition, *M. glandulosa* being the most morphologically-disparate species in clade A. Indeed, within *Macowania* as a whole, *M. glandulosa* is the only species possessing sunken leaf glands. Interestingly, *M. glandulosa* is itself disjunctly distributed, occupying two separate areas on the northeastern and southeastern arms of the Drakensberg Escarpment (Figure 1). There is some evidence of morphological divergence between the two regions, with the northernmost populations lacking glandular hairs on the leaves [80], indicating ongoing speciation in allopatry.

In contrast to clade A, adaptive divergence appears to have played a greater role in stimulating speciation in clade B. Though leaf and capitulum measurements capture the stronger morphological differentiation between species within this clade, this is apparent even on the basis of cursory visual examination. For example, striking variation is apparent in leaf morphology, involucral bract coloration (*M. revoluta* and *M. corymbosa* have brown-edged bracts) and capitulum sexuality (in contrast to the typical gynomonoecious condition, *M. revoluta* has dioecious, and *M. corymbosa*, hermaphrodite capitula). These differences are also reflected in the taxonomic history of the group, this clade containing a species which has hitherto been treated as a separate genus (*Arrowsmithia*). Within clade B, morphological differentiation is most pronounced between *M. revoluta* and *A. styphelioides*. This differentiation is almost certainly ecologically motivated, these species being fully sympatric, although they occupy different micro-habitats within their shared range (*M. revoluta* grows in deep sandy soil in bogs, while *A. styphelioides* inhabits rocky slopes).

The other sister-pair within clade B, *M. hamata* and *M. corymbosa*, shows non-overlapping ranges, occurring exclusively on the southern and northern axes of the Drakensberg, respectively. Although these species show strong morphological divergence, their habitats are similar, suggesting a scenario of adaptive divergence in allopatry.

Ecologically-driven flowering time shifts also provide a potentially important mechanism which might power genetic isolation in sympatric species. Herbarium record data reveal little to no flowering time overlap between *M. glandulosa* (October – December) and *M. sororis* (January – July)*,* and only marginal overlap between *M. glandulosa* and *M. deflexa* (December – January), suggesting that differences in phenology might influence the isolation of these species. Conversely, the data reveal that the flowering times of the sister-pair *A. styphelioides* (May – December) and *M. revoluta* (August – February) overlap; their ecological divergence thus cannot be attributed to differences in flowering time, suggesting that an alternative ecological explanation might explain their divergence in sympatry.

Although small flies have been observed visiting flowers of *Macowania* (N.Bergh, pers. obs.), little is known about the pollination mechanisms and breeding systems of these plants. Several studies (e.g. [81,82]) find pollinator diversity decreasing with increasing altitude, as well as a dominance of flies at higher altitudes. Galley et al. [16] suggest that pollinator specificity might play a role in promoting *in situ* speciation, suggesting that taxa with generalist pollination syndromes are unlikely to speciate as readily as those with specialist systems upon entering a new region. The same is suggested for *Euryops*[71], as, possibly like *Macowania,* this genus (also well-represented in the Afrotemperate regions) has a typical Asteraceae generalist pollination syndrome.

## Conclusions

We sketch a scenario of post-uplift erosion-mediated speciation in *Macowania*. Although we are aware of no studies that explore the role of erosion as a stimulus for the diversification of the Drakensberg flora, a major limitation is that the scale of speciation in many Drakensberg clades has been modest, compromising our ability to infer strong patterns. Nevertheless, these ideas could be explored in the light of phylogenetic hypotheses of the larger endemic lineages, such as *Helichrysum* and *Senecio* (Asteraceae; 29 and 22 species, respectively); *Erica* (Ericaceae; 12 species), *Delosperma* (Mesembryanthemaceae; 12 species), *Glumicalyx* (Scrophulariaceae; 6 species), *Rhodohypoxis* (Hypoxidaceae; 6 species) and *Huttonaea* (Orchidaceae; 6 species) whose radiations, like that of *Macowania*, are hypothesised to have followed major uplift at the Miocene-Pliocene boundary (e.g. [17]). These findings may indicate general patterns of diversification applicable to other tectonically-influenced systems, including the high Andes, and suggest that post-uplift habitat production by erosive processes might be as much of a driver of speciation as the initial uplift itself.

## Availability of supporting data

The datasets supporting the results of the plastid, nuclear and combined analyses is available in the TreeBASE repository, study ID 15214 http://www.treebase.org/treebaseweb/search/study/summary.html?id=15214. The Genbank accession numbers are provided in Table 1 of this manuscript.

## Abbreviations

BS: Bootstrap; PP: Posterior probability; ESS: Effective sample size; DAC: Drakensberg alpine centre.

## Competing interests

The authors declare that they have no competing interests.

## Authors’ contributions

All authors were involved in the design of the study, field collection and the analyses as well as the drafting of the paper. JB performed the majority of molecular work and phylogenetic analyses. This study formed part of the MSc thesis of JB at the University of Cape Town under the supervision of NGB and GAV. All authors read and approved the final manuscript.

## Authors’ information

Joanne Bentley is a graduate student at the Department of Biological Sciences at the University of Cape Town (UCT). She is interested in using molecular tools to examine evolutionary processes.

Tony Verboom is an Associate Professor at the Department of Biological Sciences (UCT). His interests include the evolution and diversification patterns of the Greater Cape Flora and he specialises in phylogenetic methods of analysis to answer pertinent questions.

Nicola Bergh is a Researcher at the Compton Herbarium. Her focus is the evolutionary history of paper daisies in southern Africa.

## Supplementary Material

Additional file 1 BEAST MCC tree indicating 95% HPD error bars on the nodes with a scalebar representing time in millions of years. Click here for file

## References

[B1] Simpson BB Pleistocene changes in the flora of the High Tropical Andes Paleobiology 1975 1 273 294

[B2] Burnham RJ Graham A The history of Neotropical vegetation: new developments and status Ann Mo Bot Gard 1999 86 546 589 10.2307/2666185

[B3] Hughes C Eastwood R Island radiation on a continental scale: exceptional rates of plant diversification after uplift of the Andes Proc Natl Acad Sci U S A 2006 103 10334 10339 10.1073/pnas.0601928103 16801546 PMC1502458

[B4] Bryson RW Murphy RW Lathrop A Lazcano-Villareal D Evolutionary drivers of phylogeographical diversity in the highlands of Mexico: a case study of the Crotalus triseriatus species group of montane rattlesnakes J Biogeogr 2011 38 697 710 10.1111/j.1365-2699.2010.02431.x

[B5] Bryson RW García-Vázquez UO Riddle BR Diversification in the Mexican horned lizard Phrynosoma orbiculare across a dynamic landscape Mol Phylogenet Evol 2012 62 87 96 10.1016/j.ympev.2011.09.007 21964512

[B6] Bryson RW García-Vázquez UO Riddle BR Relative roles of Neogene vicariance and Quaternary climate change on the historical diversification of bunchgrass lizards (Sceloporus scalaris group) in Mexico Mol Phylogenet Evol 2012 62 447 457 10.1016/j.ympev.2011.10.014 22075377

[B7] Xu T Abbott RJ Milne RI Mao K Du FK Wu G Ciren Z Phylogeography and allopatric divergence of cypress species (Cupressus L.) in the Qinghai-Tibetan Plateau and adjacent regions BMC Evol Biol 2010 10 194 10.1186/1471-2148-10-194 20569425 PMC3020627

[B8] Yang F-S Qin A-L Li Y-F Wang X-Q Great genetic differentiation among populations of Meconopsis integrifolia and its implication for plant speciation in the Qinghai-Tibetan Plateau PloS one 2012 7 e37196 10.1371/journal.pone.0037196 22590654 PMC3349641

[B9] Myers N Mittermeier RA Mittermeier CG Da Fonseca GA Kent J Biodiversity hotspots for conservation priorities Nature 2000 403 853 858 10.1038/35002501 10706275

[B10] Luteyn JL Churchill SP Griffin D III Gradstein SR Sipman HJM Gavilanes A A checklist of plant diversity, geographical distribution, and botanical literature 1999 New York: Botanical Garden

[B11] King LC King LA A reappraisal of the Natal monocline S Afr Geogr J 1959 41 15 30 10.1080/03736245.1959.10559341

[B12] Partridge TC Maud RR Geomorphic evolution of southern Africa since the Mesozoic S Afr J Geol 1987 90 179 208

[B13] Partridge TC Maud RR Macro-scale geomorphic evolution of southern Africa Oxf Monogr Geol Geophys 2000 40 3 18

[B14] Partridge TC Of diamonds, dinosaurs and diastrophism: 150 years of landscape evolution in southern Africa S Afr J Geol 1998 101 167 184

[B15] Carbutt C Edwards TJ The flora of the Drakensberg Alpine Centre Edinburgh J Bot 2004 60 581 607

[B16] Galley C Bytebier B Bellstedt DU Peter Linder H The Cape element in the Afrotemperate flora: from Cape to Cairo? Proc R Soc B 2007 274 535 543 10.1098/rspb.2006.0046 17476774 PMC1766381

[B17] Carlson SE Linder HP Donoghue MJ The historical biogeography of Scabiosa (Dipsacaceae): implications for Old World plant disjunctions J Biogeogr 2012 39 1086 1100 10.1111/j.1365-2699.2011.02669.x

[B18] Mcguire AF Kron KA Phylogenetic relationships of European and African Ericas Int J Plant Sci 2005 166 311 318 10.1086/427478

[B19] Goldblatt P Manning JC Plant diversity of the Cape region of South Africa Ann Mo Bot Gard 2002 89 281 302 10.2307/3298566

[B20] Killick DJB Werger MJA The Afro-alpine Region Biogeography and Ecology of Southern Africa 1978 The Hague: Springer Netherlands 515 560

[B21] Lande R Natural selection and random genetic drift in phenotypic evolution Evolution 1976 30 314 334 10.2307/2407703 28563044

[B22] Wiens JJ Speciation and ecology revisited: phylogenetic niche conservatism and the origin of species Evolution 2004 58 193 197 15058732 10.1111/j.0014-3820.2004.tb01586.x

[B23] Kozak KH Weisrock DW Larson A Rapid lineage accumulation in a non-adaptive radiation: phylogenetic analysis of diversification rates in eastern North American woodland salamanders (Plethodontidae: Plethodon) Proc R Soc B 2009 273 539 546 10.1098/rspb.2005.3326 PMC1560065 16537124

[B24] Rundell RJ Price TD Adaptive radiation, nonadaptive radiation, ecological speciation and nonecological speciation Trends Ecol Evol 2009 24 394 399 10.1016/j.tree.2009.02.007 19409647

[B25] Schluter D Ecological character displacement in adaptive radiation Am Nat 2000 156 S4 S16 10.1086/303412

[B26] Schluter D Evidence for ecological speciation and its alternative Science 2009 323 737 741 10.1126/science.1160006 19197053

[B27] Mckinnon JS Mori S Blackman BK David L Kingsley DM Jamieson L Chou J Evidence for ecology’s role in speciation Nature 2004 429 294 298 10.1038/nature02556 15152252

[B28] Orr MR Smith TB Ecology and speciation Trends Ecol Evol 1998 13 502 506 10.1016/S0169-5347(98)01511-0 21238408

[B29] Partridge TC Brink ABA Gravels and terraces of the lower Vaal River basin S Afr Geogr J 1967 49 21 38 10.1080/03736245.1967.10559417

[B30] Davies TA Hay WW Southam JR Worsley TR Estimates of Cenozoic oceanic sedimentation rates Science 1977 197 53 55 10.1126/science.197.4298.53 17828891

[B31] Watson A Williams DP Early Pleistocene river gravels in Swaziland and their geomorphological and structural significance Z Geomorphol 1985 29 71 87 New Series

[B32] Lageat Y Robb JL The relationships between structural landforms, erosion surfaces, and the geology of the Archean granite basement in the Barberton region, Eastern Transvaal Trans Geol Soc S Af 1984 87 141 159

[B33] Gilchrist AR Summerfield MA Kirkby MJ Tectonic models of passive margin evolution and their implications for theories of long-term landscape development Process models and theoretical geomorphology 1994 Chichester, UK: Wiley 55 84

[B34] Burke K Gunnell Y The African erosion surface: a continental-scale synthesis of geomorphology, tectonics, and environmental change over the past 180 million years Mem Geol Soc Am 2008 201

[B35] Linder HP The historical phytogeography of the Disinae (Orchidaceae) Bothalia 1983 14 565 570

[B36] White F Werger MJA The Afromontane region Biogeography and ecology of southern Africa 1978 The Hague: Springer Netherlands 465 510

[B37] Linder HP Meadows ME Cowling RM History of the Cape Flora The ecology of Fynbos: nutrients, fire and diversity 1992 Cape Town: Oxford University Press 113 134

[B38] Killick DJB Davis SD, Heywood VH Drakensberg Alpine Region – Lesotho and South Africa Centres of Plant Diversity 1994 Oxford: Oxford University Press 257 260

[B39] Van Wyk AE Smith GF Regions of Floristic Endemism in Southern Africa 2001 Hatfield: Umdaus Press

[B40] Carbutt C Edwards TJ The endemic and near-endemic angiosperms of the Drakensberg Alpine Centre S Afr J Bot 2006 72 105 132 10.1016/j.sajb.2005.06.001

[B41] Kroner G Systematische Studien im Umkreis von Athrixia Ker-Gawler (Asteraceae) Mitt Bot St Samml Munchen 1980 16 1 267

[B42] Hilliard OM Burtt B Notes on some plants of southern Africa Notes Roy Bot Gard Edinburgh 1985 42 230 233

[B43] Bergh NG Linder HP Cape diversification and repeated out-of-southern-Africa dispersal in paper daisies (Asteraceae-Gnaphalieae) Mol Phylogenet Evol 2009 51 5 18 10.1016/j.ympev.2008.09.001 18822381

[B44] Ward J Bayer RJ Breitwieser I Smissen R Galbany-Casals M Unwin M Funk VA, Susanna A, Stuessy TF, Bayer RJ Ch. 36: Gnaphalieae Systematics, Evolution and Biogeography of Compositae 2009 Vienna: International Association for Plant Taxonomy 539 588

[B45] Galbany-Casals M Andrés-Sánchez S Garcia-Jacas N Susanna A Rico E Montserrat Martínez-Ortega M How many of Cassini anagrams should there be? Molecular systematics and phylogenetic relationships in the *Filago* group (Asteraceae, Gnaphalieae), with special focus on the genus *Filago* Taxon 2010 59 1671 1689

[B46] Bergh NG Trisos CH Verboom GA Phylogeny of the “*Ifloga* clade” (Asteraceae, Gnaphalieae), a lineage occurring disjointly in the Northern and Southern Hemisphere, and inclusion of *Trichogyne* in synonymy with *Ifloga* Taxon 2011 60 1065 1075

[B47] Doyle J Doyle JL Genomic plant DNA preparation from fresh tissue-CTAB method Phytochem Bull 1987 19 11 15

[B48] Gavel NJ Jarrett RL A modified CTAB DNA extraction procedure for *Musa* and *Ipomoea* Plant Mol Biol Rep 1991 9 262 266 10.1007/BF02672076

[B49] Bayer RJ Puttock CF Kelchner SA Phylogeny of South African Gnaphalieae (Asteraceae) based on two noncoding chloroplast sequences Am J Bot 2000 87 259 272 10.2307/2656914 10675314

[B50] Baldwin BG Markos S Phylogenetic utility of the external transcribed spacer (ETS) of 18S-26S rDNA: congruence of ETS and ITS trees of Calycadenia (Compositae) Mol Phylogenet Evol 1998 10 449 463 10.1006/mpev.1998.0545 10051397

[B51] Markos S Baldwin BG Higher-level relationships and major lineages of *Lessingia* (Compositae, Asteraceae) based on nuclear rDNA internal and external transcribed spacer (ITS and ETS) sequences Syst Biol 2001 26 168 183

[B52] White TJ Bruns T Lee S Taylor JW Innis MA, Gelfand DH, Sninsky JJ, White TJ Amplification and direct sequencing of fungal ribosomal RNA genes for phylogenetics PCR Protocols: A Guide to Methods and Applications 1990 New York: Academic Press Inc 315 322

[B53] Taberlet P Gielly L Pauton G Bouvet J Universal primers for amplification of three non-coding regions of chloroplast DNA Plant Mol Biol 1991 17 1105 1109 10.1007/BF00037152 1932684

[B54] Sang T Crawford DJ Stuessy TF Chloroplast DNA phylogeny, reticulate evolution, and biogeography of *Paeonia* (Paeoniaceae) Am J Bot 1997 84 1120 1136 10.2307/2446155 21708667

[B55] Hall TA BioEdit: a user-friendly biological sequence alignment editor and analysis program for Windows 95/98/NT Nucleic Acids Symp 1999 41 95 98

[B56] Felsenstein J Confidence limits on phylogenies: an approach using the bootstrap Evolution 1985 39 783 791 10.2307/2408678 28561359

[B57] Swofford DL PAUP*. Phylogenetic analysis using parsimony (*and other methods). Version 4 1998 Sunderland MA: Sinauer Associates

[B58] Felsenstein J Cases in which parsimony or compatibility methods will be positively misleading Syst Zool 1978 27 401 410 10.2307/2412923

[B59] Alfaro ME Holder MT The posterior and the prior in Bayesian phylogenetics Annu Rev Ecol Evol Syst 2006 37 19 42 10.1146/annurev.ecolsys.37.091305.110021

[B60] Hillis DM Bull JJ An empirical test of bootstrapping as a method for assessing confidence in phylogenetic analyses Syst Biol 2009 59 182 192

[B61] Huelsenbeck JP Ronquist F MRBAYES: Bayesian inference of phylogenetic trees Bioinformatics (Oxford, England) 2001 17 754 755 10.1093/bioinformatics/17.8.754 11524383

[B62] Nylander JAA MrModeltest v2 Programme distributed by author 2004 Evolutionary Biology Centre, Uppsala University 2 [http://www.abc.se/~nylander/mrmodeltest2/mrmodeltest2.html]

[B63] Akaike H A new look at the statistical model identification IEEE Trans Automat Contr 1994 19 716 723

[B64] Drummond AJ Rambaut A BEAST: Bayesian evolutionary analysis by sampling trees BMC Evol Biol 2007 7 214 10.1186/1471-2148-7-214 17996036 PMC2247476

[B65] Graur D Martin W Reading the entrails of chickens: molecular timescales of evolution and the illusion of precision Trends Genet 2004 20 80 86 10.1016/j.tig.2003.12.003 14746989

[B66] Calenge C The package “adehabitat” for the R software: a tool for the analysis of space and habitat use by animals Ecol Model 2006 197 516 519 10.1016/j.ecolmodel.2006.03.017

[B67] Burgman MA Fox JC Bias in species range estimates from minimum convex polygons: implications for conservation and options for improved planning Anim Conserv 2003 6 19 28 10.1017/S1367943003003044

[B68] Fitzpatrick BM Turelli M The geography of mammalian speciation: mixed signals from phylogenies and range maps Evolution 2006 60 601 615 16637504

[B69] Yates MJ Verboom GA Rebelo AG Cramer MD Ecophysiological significance of leaf size variation in Proteaceae from the Cape Floristic Region Funct Ecol 2010 24 485 492 10.1111/j.1365-2435.2009.01678.x

[B70] Mahalanobis PC On the generalized distance in statistics Proc Nat Instit Sci India 1936 2 49 55

[B71] Devos N Barker NP Nordenstam B Mucina L A multilocus phylogeny of *Euryops* (Asteraceae: Senecioneae) augments support for the “Cape to Cairo” hypothesis of floral migrations in Africa Taxon 2010 59 57 67

[B72] Wolfenden E Ebinger C Yirgu G Deino A Ayalew D Evolution of the northern Main Ethiopian rift: birth of a triple junction Earth Planet Sc Lett 2004 224 213 228 10.1016/j.epsl.2004.04.022

[B73] Chorowicz J The East African rift system J Afr Earth Sci 2005 43 379 410 10.1016/j.jafrearsci.2005.07.019

[B74] Sepulchre P Ramstein G Fluteau F Schuster M Tiercelin J-J Brunet M Tectonic uplift and Eastern Africa aridification Science 2006 313 1419 1423 10.1126/science.1129158 16960002

[B75] Sakai T Saneyoshi M Tanaka S Sawada Y Nakatsukasa M Mbua E Ishida H Climate shift recorded at around 10 Ma in Miocene succession of Samburu Hills, northern Kenya Rift, and its significance Geol Soc London Spec Publ 2010 342 109 127 10.1144/SP342.9

[B76] Levin NE Quade J Simpson SW Semaw S Rogers M Isotopic evidence for Plio-Pleistocene environmental change at Gona, Ethiopia Earth Planet Sc Lett 2004 219 93 110 10.1016/S0012-821X(03)00707-6

[B77] Cerling TE Harris JM Macfadden BJ Leakey MG Quadek J Eisenmann V Ehleringer JR Global vegetation change through the Miocene/Pliocene boundary Nature 1997 389 153 158 10.1038/38229

[B78] Moore AE Cotterill FPDW Main MPL Williams HB Gupta A The Zambezi River Large Rivers: Geomorphology and Management 2007 England: Wiley 311 332

[B79] Mucina L Rutherford MC Mucina L, Rutherford MC The vegetation of South Africa, Lesotho and Swaziland Strelitzia 19 2006 Pretoria: South African National Biodiversity Institute 585 614

[B80] Hilliard OM Burtt B Macowania Notes Roy Bot Gard 1976 34 260 279

[B81] Arroyo MTK Primack R Armesto J Community studies in pollination ecology in the high temperate Andes of Central Chile. I. Pollination mechanisms and attitudinal variation Am J Bot 1982 69 82 97 10.2307/2442833

[B82] Elberling H Olesen JM The structure of a high latitude plant-flower visitor system: the dominance of flies Ecography 1999 22 314 323 10.1111/j.1600-0587.1999.tb00507.x

