# Supplementary figures and images for: Erosive processes after tectonic uplift stimulate vicariant and adaptive speciation: evolution in an Afrotemperate-endemic paper daisy genus
Source: BMC Evol Biol. 2014 Feb 13;14:27. doi: 10.1186/1471-2148-14-27 (PMC3927823; doi:10.1186/1471-2148-14-27)

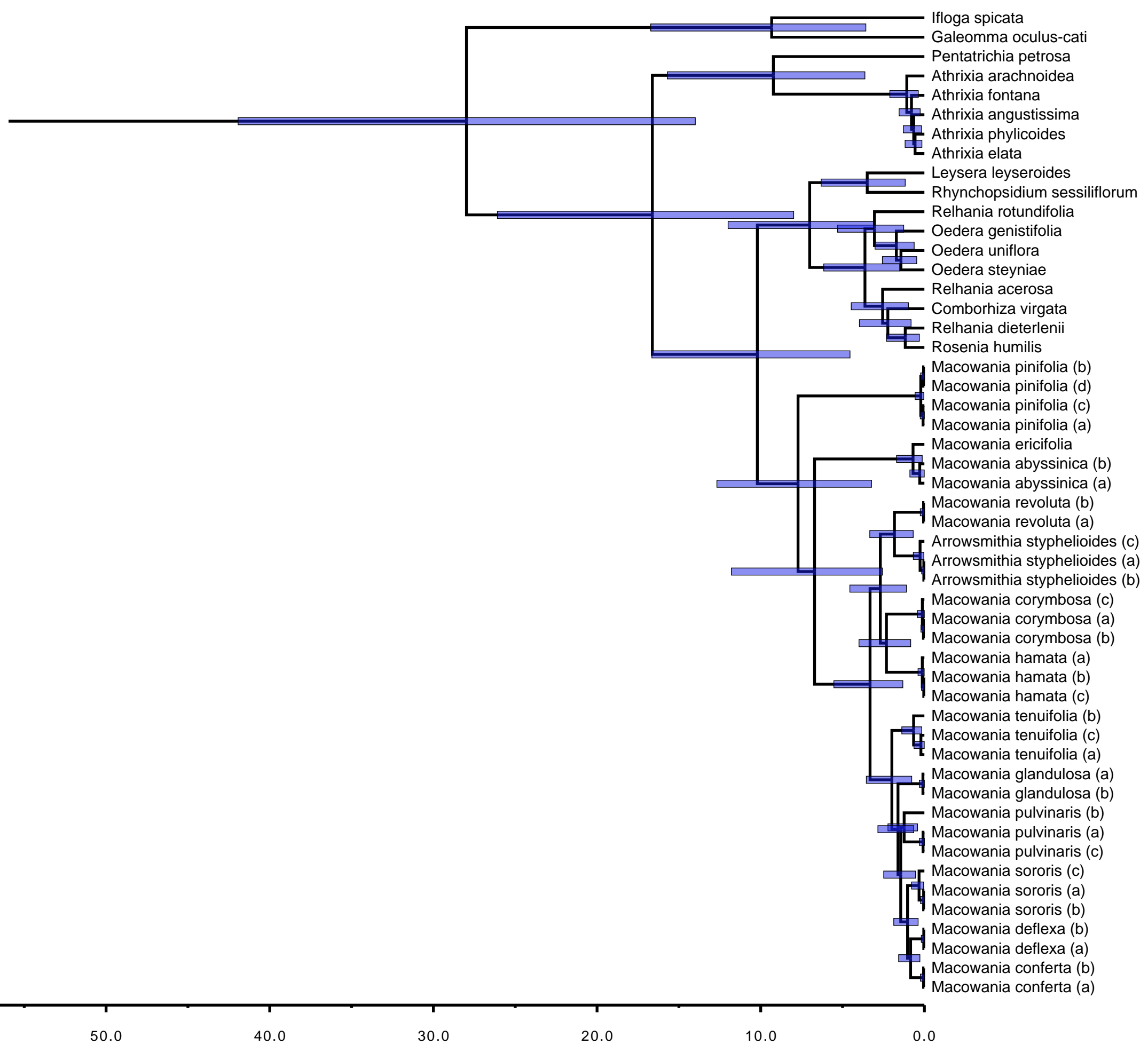

Supplement: Additional file 1 — BEAST MCC tree indicating 95% HPD error bars on the nodes with a scalebar representing time in millions of years. [file 1471-2148-14-27-S1.pdf]
